# Development of Magnetizable, Nickel–Ferrite-Decorated Carbon Nanocomposites as Hydrogenation Catalyst for Aniline Synthesis

**DOI:** 10.3390/ijms242417547

**Published:** 2023-12-16

**Authors:** Ádám Prekob, Máté Péter Szegedi, Gábor Muránszky, Ferenc Kristály, Miklós Nagy, Gyula Halasi, Ákos Szamosvölgyi, Béla Fiser, Béla Viskolcz, László Vanyorek

**Affiliations:** 1 Institute of Chemistry, University of Miskolc, Miskolc-Egyetemváros, 3515 Miskolc, Hungary; adam.prekob@uni-miskolc.hu (Á.P.); mate.peter.szegedi@uni-miskolc.hu (M.P.S.); gabor.muranszky@uni-miskolc.hu (G.M.); miklos.nagy@uni-miskolc.hu (M.N.); bela.viskolcz@uni-miskolc.hu (B.V.); 2 Institute of Mineralogy and Geology, University of Miskolc, Miskolc-Egyetemváros, 3515 Miskolc, Hungary; askkf@uni-miskolc.hu; 3 Department of Applied and Environmental Chemistry, University of Szeged, Rerrich Béla Square 1., 6720 Szeged, Hungary; halasigy@chem.u-szeged.hu (G.H.); szamosvolgyi@chem.u-szeged.hu (Á.S.); 4 ELI-ALPS, ELI-HU Non-Profit Ltd., Wolfgang Sandner Utca 3., H-6728 Szeged, Hungary; 5 Higher Education and Industrial Cooperation Centre, University of Miskolc, Miskolc-Egyetemváros, 3515 Miskolc, Hungary; 6 Ferenc Rakoczi II Transcarpathian Hungarian College of Higher Education, 90200 Beregszász, Ukraine; 7 Department of Physical Chemistry, Faculty of Chemistry, University of Lodz, 90-236 Lodz, Poland

**Keywords:** nitrobenzene, magnetic nanoparticles, carbon nanolayers, stability

## Abstract

Catalysts with magnetic properties can be easily recovered from the reaction medium without loss by using a magnetic field, which highly improves their applicability. To design such systems, we have successfully combined the magnetic properties of nickel ferrite nanoparticles with the positive properties of carbon-based catalyst supports. Amine-functionalized NiFe_2_O_4_ nanoparticles were deposited on the surfaces of nitrogen-doped bamboo-like carbon nanotubes (N-BCNT) and carbon nanolayers (CNL) by using a coprecipitation process. The magnetizable catalyst supports were decorated by Pd nanoparticles, and their catalytic activity was tested through the hydrogenation of nitrobenzene (NB). By using the prepared catalysts, high nitrobenzene conversion (100% for 120 min at 333 K) and a high aniline yield (99%) were achieved. The Pd/NiFe_2_O_4_-CNL catalyst was remarkable in terms of stability during the reuse tests due to the strong interaction formed between the catalytically active metal and its support (the activity was retained during four cycles of 120 min at 333 K). Furthermore, despite the long-lasting mechanical stress, no significant palladium loss (only 0.08 wt%) was detected.

## 1. Introduction

Aniline is one of the main precursors of polyurethanes [1]. It is produced in industrial quantities via the catalytic hydrogenation of nitrobenzene. Thus, proper catalysts are needed for its production. Carbon-supported catalysts are the most commonly used ones in hydrogenation reactions due to their high specific surface area, chemical resistance, and easy and inexpensive accessibility [2]. Furthermore, carbon materials are available in many different forms with varied properties, such as nanotubes, nanofibers, and different activated carbon structures [3,4,5,6]. 

An interesting carbon structure can be prepared with the modification of the well-known demonstration experiment called “Sugar snake”. In this reaction, a carbon source (sugar) is reacted with sulfuric acid, producing a foam-like carbon form. This carbon foam has already been used in electrocatalytic reactions several times [7,8,9]. It is decorated by Co or Fe nanoparticles and used in fuel cells and metal–air batteries. Using 4-nitroaniline instead of sugar and applying an elevated temperature, a N-doped version of the foam can be prepared.

Carbon nanotubes (CNTs) have a wide range of applications, including biosensors, electrodes, mechanical structures, and catalyst carriers [10,11,12,13], due to their excellent electric conductivity, mechanical resistance, heat conductivity, and surface functionality [14,15,16,17,18]. Their chemical resistance and mechanical and thermal stability make them exceptional catalyst carriers, and they have already been used in many catalytic reactions [19,20,21].

To further develop the properties of carbon carriers, doping can be used (e.g. nitrogen doping) where heteroatoms are incorporated into the system, resulting a distorted structure [22,23,24]. Distorted structures contain defects (e.g., vacancies) in the crystal structure, which are ideal binding sites for different molecules or catalytically active metal particles. In situ nitrogen doping can be easily achieved by using a nitrogen-containing carbon source for the reaction. The advantageous effects of N-doped catalysts have already been proven in many different hydrogenation reactions, like aldehyde, ester, nitrophenol, cellobiose, or nitrobenzene hydrogenation [25,26,27,28,29].

Although the use of carbon carriers is advantageous, their recoverability is difficult and often causes catalyst loss, since the small particles are difficult to filter and settle. However, by combining carbon carriers with magnetic particles, the catalytic system could be easily separated while the carbon continues to exert its beneficial effects. A similar magnetically retrievable system was prepared by Akbarzadeh et al., in which magnetic nanoparticles were synthetized on the surfaces of carbon nanotubes [30]. The composite was used for the synthesis of isochromeno [4,3-c]pyrazole-5(1H)-one, providing a product yield of more than 90%.

Ferrites are commonly used catalyst carriers [31,32] and catalysts [33,34] in different organic and inorganic reactions. Nickel–ferrite supported catalyst was tested by Hajdu et al. for the hydrogenation of nitrobenzene and dinitrotoluene [35,36]. The catalysts showed high activity and product yield in both cases.

In this paper, the preparation of special magnetic carbon composite catalyst carriers is presented by using two different nitrogen-doped carbon forms (carbon nanolayers (CNL) and nitrogen-doped bamboo-like carbon nanotubes (N-BCNT)). The carriers were decorated with Pd nanoparticles, characterized in detail with electron microscopy and X-ray diffraction, and tested by nitrobenzene hydrogenation.

## 2. Results and Discussion

### 2.1. Characterization of the CNL and N-BCNT Nanomaterials as Catalyst Supports

**Figure 1:**
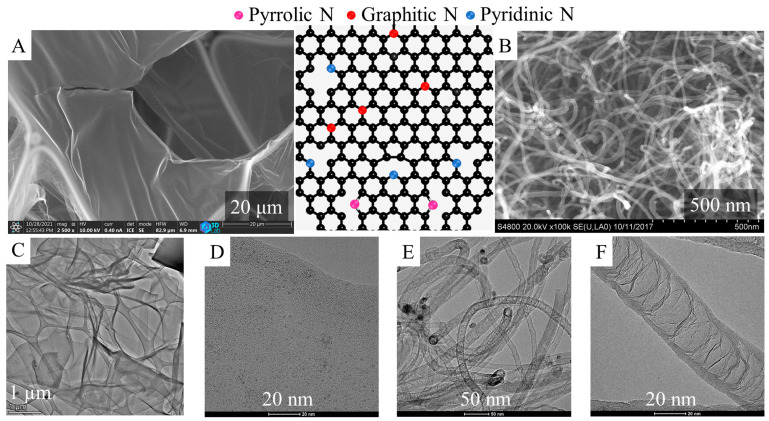
SEM and HRTEM images of the carbon nanolayers (CNL) (**A**,**C**,**D**) and nitrogen-doped carbon nanotubes (N-BCNT) (**B**,**E**,**F**).

The applied carbon nanolayers were prepared by using 4-nitroaniline as a carbon source in a reaction similar to the “sugar snake” experiment, then examined by SEM and HRTEM, which verified the membranous foil-like structure of the carbon form (Figure 1A–D). The foils were built up by multiple atomic layers, creating an N-doped carbon structure a few nanometers thick. These foils were stretched out between thicker carbon structures, which served as a kind of frame. It can be seen in the images that the membrane-like layers were crumbled in some places, or even torn. These nitrogen-doped carbon foils were advantageous supports for the palladium nanoparticles during the preparation of the catalyst, because on the surfaces of these layers, the nanoparticles could be efficiently anchored. Moreover, the catalytically active metal particles were located on the surfaces of the carbon nano-foils, which were easily accessible for reactant molecules; therefore, the catalytic reaction was faster than in the case of the porous catalysts. Similar advantageous properties were present in the morphology of the N-BCNTs (Figure 1 B,E,F). Another important feature of the CNL and N-BCNTs was that these were nitrogen-doped carbon forms, so they incorporated nitrogen atoms into their structure, such as pyridinic, pyrrolic, and graphitic nitrogen types [22]. The incorporation of nitrogen atoms (mainly the pyrrolic and pyridinic types) in the honeycomb-like lattice led to structural defects (vacancies, bonding disorders, non-cyclized structures), which caused a high level of disorder in the structure. This provided additional interaction points for the catalytically active metal particles. Due to the incorporation of nitrogen atoms, half-fullerene-like caps were formed from the carbon atoms, from which the special bamboo-like structure was also built up (Figure 1F). At the edges of the mentioned half-fullerene-like caps, sp3 carbon atoms were located, to which several oxygen containing functional groups were attached. This further improved the adsorption of the catalytically active metal ions (by electrostatic interaction or ion exchange) [37,38].

**Figure 2:**
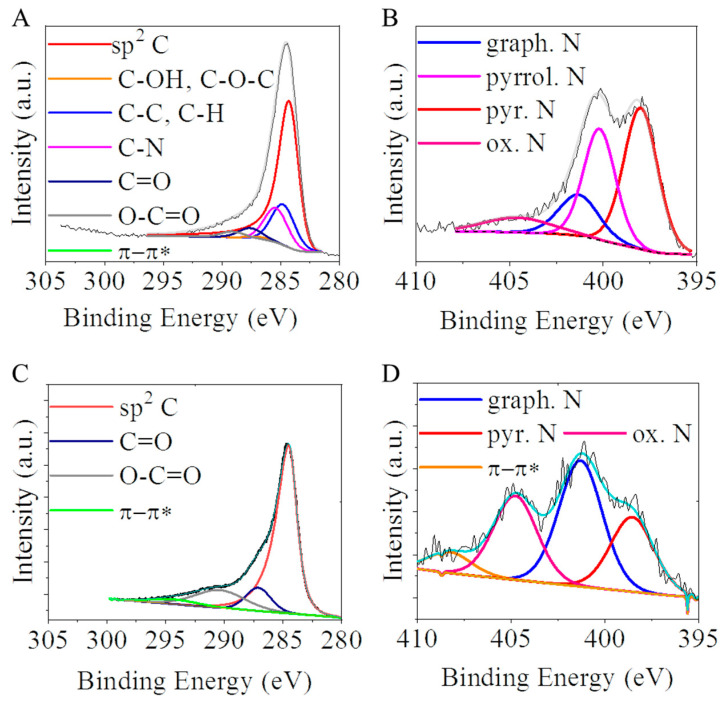
XPS spectrum with the deconvoluted C 1s and N 1s bands of the carbon nanolayer (**A**,**B**) and N-BCNT (**C**,**D**) samples.

Oxygen-containing functional groups, namely, the -OH and -COOH, were identified by X-ray photoelectron spectroscopy (XPS) on the carbon supports. Their presence is highly beneficial, because via deprotonation, the surface charge can be altered to be more negative, contributing to the anchoring of the catalytically active metal ions (i.e., Pd^2+^) on the catalyst supports during the preparation of the catalyst [37,38]. These groups were identifiable by the evaluation of the C 1s peak on the XPS spectrum (Figure 2A,C). On the deconvoluted C 1s band of the CNL and N-BCNTs, the main peaks were seen at 284.5 eV, with high intensity. These could be assigned to the sp^2^ carbon atoms, part of the graphitic regions, and this demonstrates that most of the C atoms were arranged in a honeycomb lattice in the carbon layer of CNL and in the fibrous structure of N-BCNTs. On the deconvoluted C 1s band of the carbon nanolayers, five more weak peaks centered at 284.9, 285.5, 287.2, 287.8, and 289.2 eV binding energy, corresponding to the C-C or C-H; C-N, C-OH, and C-O-C; C=O, C-N or C-O; C=O; and O=C-O groups were formed during the oxidation step (in CO_2_ atmosphere at 900 °C) during the activation. In the case of the N-BCNTs, after deconvoluting the C 1s band only, two peaks could be identified next to the sp^2^ carbons at 287.2 and 290.5 eV; these binding energies were characteristic of the carbonyl (C=O) and carboxyl (O-C=O) groups. On the surfaces of N-BCNTs, fewer oxygen-containing functional groups formed, unlike in the case of the carbon nanolayer sample. The total oxygen content of the N-BCNT was 2.6 atomic % significantly lower than in case of the CNL (5.9 at%).

As mentioned previously, the carboxyl- and hydroxyl functional groups on the surface of the supports were able to deprotonate in aqueous medium, leading to a negative surface charge. To verify this, zeta potential measurements were carried out, and it was found that the electrokinetic potentials were similar in the case of the two studied carbon supports: −24.8 mV (CNL and −21.9 mV (N-BCNT) (Supplementary Information Figure S1). Due to the negative surface charge, carbon surfaces were well dispersed and easily accessible for the catalytically active metal ions, which could be anchored on the surface by ion exchange adsorption or electrostatic interaction. Thus, homogenous coverage of the surface with catalytically active metal ions or metal nanoparticles could be achieved. Moreover, on polar surfaces, the polar reactant molecules adsorbed in greater quantities, which is another key step in catalytic reactions.

The two carbon forms contained incorporated nitrogen atoms in their structures because they were prepared from nitrogen-containing precursors (*n*-butyl amine and *p*-nitro aniline). Thus, the N 1s band could also be found on the XPS spectrum of both of the CNL and N-BCNT. The N 1 s peak of the carbon nanolayer could be deconvoluted into four individual peaks located at 398.0 eV, 400.2 eV, 401.4 eV, and 404.6 eV which can be associated with pyridinic, pyrrolic, graphitic, and oxidized nitrogen, respectively (Figure 2B). On the N 1s band of the N-BCNT, the peaks of the pyridinic (398.6 eV), graphitic (401.3 eV) and oxidized nitrogen (404.8 eV) were found (Figure 2D). According to the XPS elemental analysis, the amounts of nitrogen in the N-BCNT and CNL were 3.2 at% and 13.4 at%, respectively. The nitrogen atoms incorporated in the structure of the N-BCNT and CNL had significant influence on the structures of these nano carbon-forms, because the bond lengths of C-N (1.41 Å) and C-C (1.42 Å) in the cases of pyridinic and graphitic N bonds were similar. However, the pyrrolic N with sp^3^ bond disrupted the six-atom ring structure of the aforementioned carbon forms. Another effect of the pyridinic nitrogen formation is that monovacancies are also formed as defects in the carbon structure. In this sense, nitrogen atoms in the honeycomb-like lattice are accompanied by structural defects (vacancies, bonding disorders, non-cyclized structures, etc.) which creates a high level of disorder in the structure. Moreover, due to the high electronegativity and smaller covalent radius of nitrogen, the doping would significantly influence the structure and electronic properties of the foil-like structure of the carbon nanolayers and N-BCNT. This is also favorable for anchoring the catalytically active metals (i.e., palladium) and transition metal oxides (ferrites) around the defects and sites of the doped N atoms [39]. An increased amount of pyridinic nitrogen was found in the structure of the carbon nanolayers (Table 1). Moreover, the incorporation of nitrogen as pyrrolic binding type was observed only in the case of CNL. In this sense, a higher number of vacancies could form in the structure compared to the N-BCNT. The nitrogen doping of carbon would also lead to higher selectivity. An example for this can be found in the literature regarding heterogeneous hydrogenation reactions. Higher selectivity was reported towards aminobenzaldehyde by using a Pd/N-BCNT catalyst in the hydrogenation of nitrobenzaldehyde, which was majorly attributed to the presence of Pd-N complexes. These surface Pd-N complexes decrease the sintering of the palladium nanoparticles, thus improving the stability and the dispersion of the Pd particles [40]. The survey and O1s spectra can be found in the Supplementary Information (Supplementary Information Figure S2).

### 2.2. Characterization of the Magnetizable, Nickel-Ferrite Decorated Carbon Nanomaterials and the Palladium Catalyst

**Figure 3:**
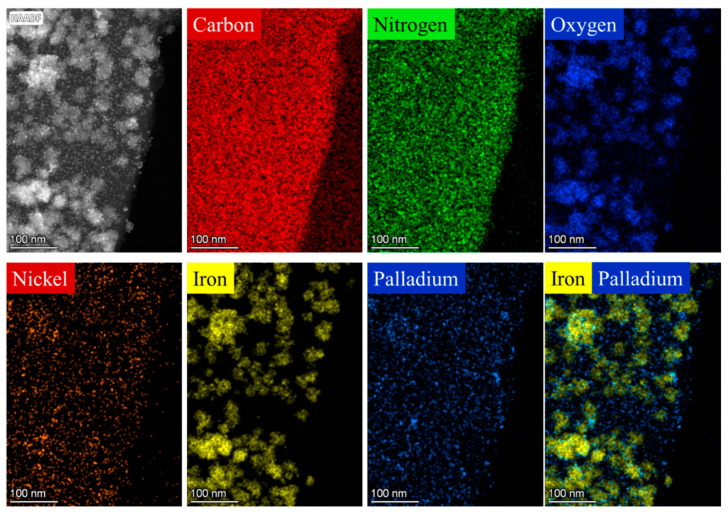
Element mapping of the Pd/NiFe_2_O_4_-CNL catalyst.

The two different carbon-supported magnetic palladium catalysts were examined by HRTEM. On the HRTEM images, the aggregated nickel ferrite particles and nanoparticles with small particle sizes can be seen (Supplementary Information Figure S3). The aggregated NiFe_2_O_4_ spheres were built up from small (about 4–7 nm) nanoparticles (Supplementary Information Figure S4). These nanospheres were 26 ± 6 nm and 40 ± 13 nm in diameter in the cases of the NiFe_2_O_4_-CNL and NiFe_2_O_4_-N-BCNT systems, respectively (Supplementary Information Figure S5). The surfaces of the carbon supports were richly coated by nanoparticles. However, the palladium nanoparticles were not visually distinguishable from the nickel–ferrite nanoparticles due to their small particle sizes; thus, element mapping was employed to characterize the orientation of Pd particles on the catalyst surface (Figure 3). In the high-angle annular dark-field (HAADF) TEM images, the characteristics of NiFe_2_O_4_ aggregates could be found, which were built up from small ferrite particles. Furthermore, on the surfaces of carbon supports, very small nanoparticles were also visible; these were palladium crystallites, based on their morphological characteristics. It is important to determine which surface is preferred by palladium particles—the carbon surface or the oxide surface of the spinel. The promoter effect of ferrites can take effect if there is direct contact between the noble metal and the oxide phase, which was also studied by element mapping. It was found that the Pd nanoparticles were present on the entire surface of the catalyst, including the surfaces of the ferrite particles. This phenomenon was due to the presence of several -NH_2_ and oxygen-containing functional groups (-OH and -COOH); moreover, the structural defects also contributed to the efficient anchoring of Pd crystallites. The high nitrogen content can be explained by the presence of incorporated N atoms and surface -NH_2_ groups. The oxygen signal was the strongest in the immediate vicinity of the ferrites. It was observed that the nickel particles accumulated in an area where the presence of iron could not be detected on the surface. Nickel nanoparticles were also present in the catalyst, and thus could act as promoters. In the case of alloy formation, electron transfer was present between the palladium and nickel, which modified the catalytic activity of the palladium. The electron structure of the Pd could overlap with that of the nickel, causing significant modifications upon bonding due to differences in the electronegativity of the two metals. This phenomenon caused electron transfer from Ni to Pd, thereby forming electron-rich Pd centers [41]. On such electron-rich palladium surfaces, the dissociation of the hydrogen molecules is more efficient. Therefore, the synergic effect between the Ni and Pd particles provided a significant increase in terms of catalyst stability, activity, and aniline selectivity during the hydrogenation tests [42].

**Figure 4:**
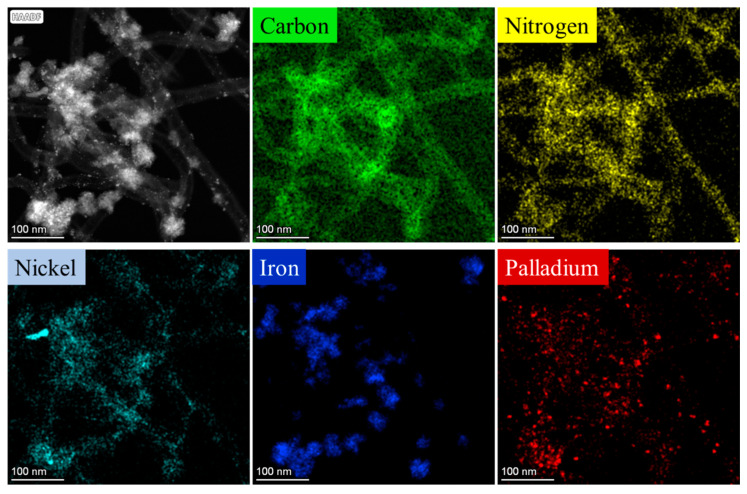
Element mapping of the Pd/NiFe_2_O_4_-N-BCNT catalyst.

This was also confirmed in the case of the Pd/NiFe_2_O_4_-N-BCNT system with respect to the location of palladium and nickel particles on the catalyst surface (Figure 4). The palladium particles were also visible on the surfaces of N-BCNTs and the ferrites. The nickel was found on the N-BCNT surface as individual metallic phase nanoparticles. However, it must be noted that the presence of nickel could have also originate from the Ni/MgO catalyst applied during the synthesis of the N-BCNT.

**Figure 5:**
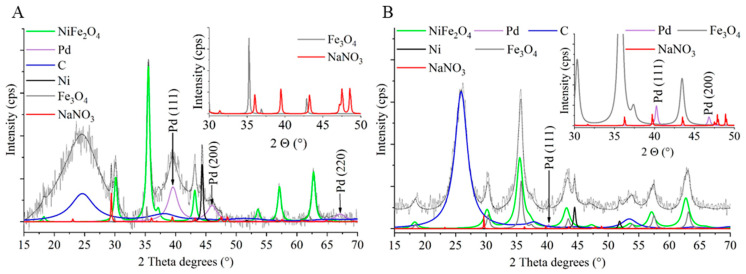
XRD patterns of the Pd/NiFe_2_O_4_-CNL (**A**) and Pd/NiFe_2_O_4_-N-BCNT (**B**) catalysts.

XRD measurements were carried out to identify the spinel structure of the prepared magnetic particles (Figure 5A,B). In the diffractogram, the (111), (220), (311), (222), (400), (422), (511), and (440) reflexions are identified at 18.1°, 30.1°, 35.5°, 37.2°, 43.2°, 53.8°, 57.2°, and 62.8° two theta degrees, which belonged to the NiFe_2_O_4_ spinel (PDF 10-0325). The characteristic reflexions of the magnetite were identified at 18.3° (111), 30.1° (220), 35.3° (311), 42.8° (400), 53.5° (422), 57.1° (511), and 62.3° (440), with two theta degrees (PDF 19-629). The (002) and (100) reflexions of the carbon nanolayer support were also found. Moreover, elemental nickel was identified in the catalyst; its (111) and (200) reflexions were found at 44.3° and 51.7°, with two theta degrees (PDF 04-0850). The nickel nanoparticles are also found upon element mapping (Figure 3 and Figure 4). Reflexions of the sodium nitrate were also found in the catalyst sample, at 23.0° (012), 29.4° (104), 31.4° (006), 35.7° (110), 39.5° (113), 43.3° (202), 47.5° (018), and 48.6° (116) degrees (PDF: 00-036-1474). The presence of the sodium nitrate phase can be explained by the reaction between the Na^+^ ions (from the sodium acetate) and NO_3_^−^ ions (from the iron and nickel precursors) during the synthesis of the nickel–ferrite nanoparticles. In addition to these phases, elemental palladium could also be identified on the diffractograms. The (111), (200), and (220) reflexions of the elemental palladium were located at 39.6°, 46.1°, and 67.2 °, with two theta degrees, respectively, in the case of all catalysts (PDF 46-1043). In the case of the N-BCNT-supported magnetic catalyst, the phases detailed above were also identified (Figure 5B).

**Figure 6:**
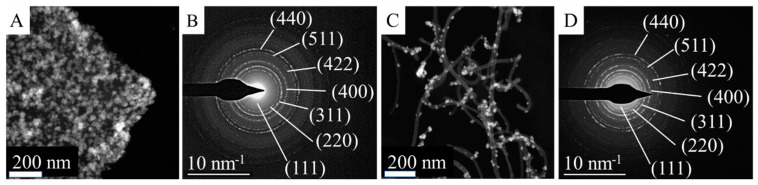
High-angle, annular, dark-field TEM images of Pd/NiFe_2_O_4_-CNL (**A**) and Pd/NiFe_2_O_4_-N-BCNT (**C**), selected-area electron diffraction of Pd/NiFe_2_O_4_-CNL (**B**) and Pd/NiFe_2_O_4_-N-BCNT (**D**) systems.

Based on the results of the powder diffraction, the phase composition of the two catalyst samples was identified. The individual aggregated NiFe_2_O_4_ particles on the carbon surface were examined by selected area electron diffraction (SAED) measurements. Based on the measured d spacing, which was correlated with d-values in X-ray databases, the aggregated particles were identified as nickel–ferrite (Figure 6).

The palladium content of the freshly prepared catalysts was measured by ICP-OES. It was found to be 3.98 wt% for the Pd/NiFe_2_O_4_-CNL and 4.26 wt% for the Pd/NiFe_2_O_4_-N-BCNT system. One of the most important parameters of the support is its specific surface area, which can influence the adsorption capacity of the catalyst. Therefore, the surface areas of the Pd/NiFe_2_O_4_-CNL and Pd/NiFe_2_O_4_-N-BCNT catalytic systems were determined. In the case of the carbon foil-based catalyst, the surface was 189 m^2^/g, while in case of the N-BCNT containing system, it was lower, only 146 m^2^/g. The sorption isotherms are provided in the Supplementary Information Figure S6.

The nickel–ferrite and magnetite contents were measured by XRD (Table 2).

**Figure 7:**
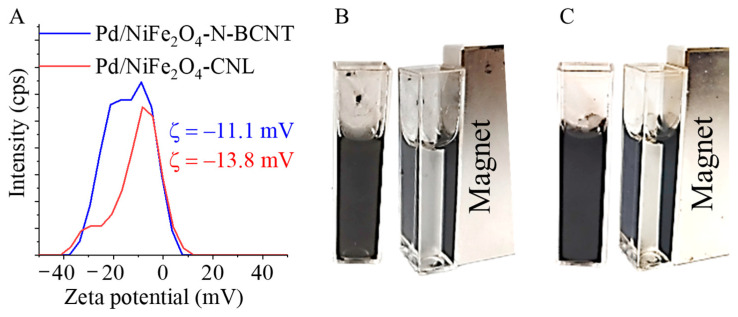
Zeta potential distribution (**A**) and dispersibility and magnetic separability of the Pd/NiFe_2_O_4_-CNL (**B**) and Pd/NiFe_2_O_4_-N-BCNT (**C**) catalysts.

The surface functional groups, carboxyl-, hydroxyl-, and amine, together changed the zeta potential of the catalysts. After the formation of the -NH_2_ groups during the preparation of the catalyst, we noticed a shift in zeta potential towards less negative values: −11.0 mV (Pd/NiFe_2_O_4_-CNL) and −13.8 mV (Pd/NiFe_2_O_4_-N-BCNT) (Figure 7A). Even though the electrokinetic potential was less negative compared to empty carbon supports, the catalysts were still well-dispersed in polar media (Figure 7B,C). Furthermore, owing to the presence of magnetic nanoparticles, the Pd/NiFe_2_O_4_-CNL and Pd/Fe_2_O_4_-N-BCNT catalysts were easily separable from the liquid phase by a magnetic field. This useful feature allows for an easy and fast recovery from the products, and, thus, using filtering technologies is not necessary.

### 2.3. Catalytic Tests of the Magnetic Carbon-Supported Palladium Catalysts in Nitrobenzene Hydrogenation

**Figure 8:**
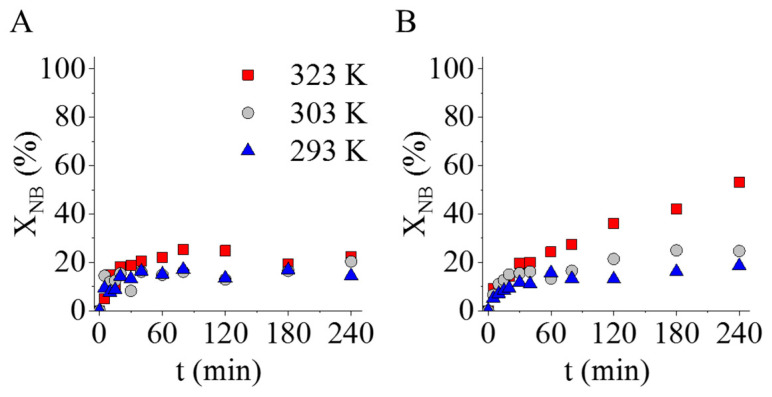
Tests of the palladium-free, nickel–ferrite-decorated CNL (**A**) and N-BCNT (**B**) catalyst supports.

The two prepared catalysts were tested in aniline synthesis at three different reaction temperatures. Before the formal catalytic measurements, the nickel–ferrite-decorated carbon supports without palladium were also tested in nitrobenzene (NB) hydrogenation at 293 K, 303 K, and 323 K. The catalytic tests confirmed that the palladium-free magnetic catalyst supports were also catalytically active, because 23 n/n% and 53 n/n% of nitrobenzene converted after 240 min of hydrogenation (Figure 8). In the case of the NiFe_2_O_4_-N-BCNT sample, higher NB conversion (53 n/n%) was observed at 323 K compared to the NiFe_2_O_4_-CNL support (23 n/n%). Although the catalytic activity was verified, the measured nitrobenzene conversion was too low in both cases; thus, palladium was used to increase the catalytic activity.

**Figure 9:**
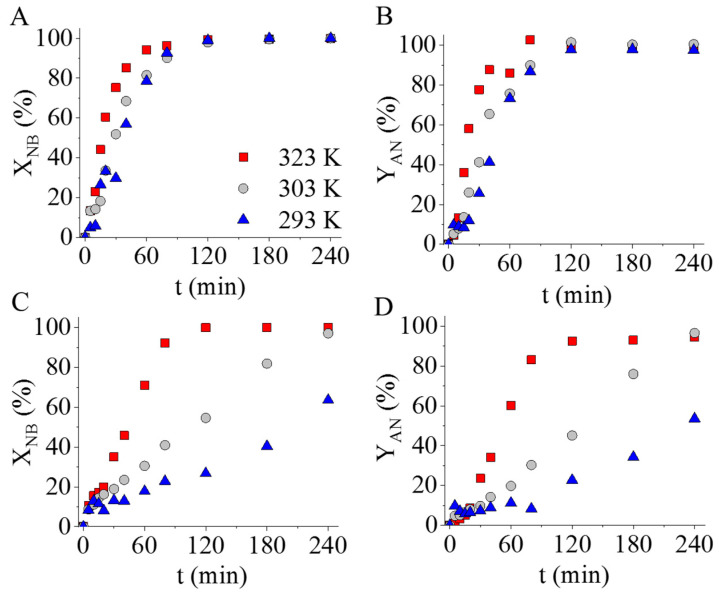
Nitrobenzene conversion and aniline yield vs. time of hydrogenation using Pd/NiFe_2_O_4_-CNL (**A**,**B**) and Pd/NiFe_2_O_4_-N-BCNT (**C**,**D**) catalysts.

The palladium-containing catalysts showed high catalytic activity at a higher (at 323 K) reaction temperature (Figure 9). The Pd/NiFe_2_O_4_-CNL-supported catalyst was more active, as was indicated by the fact that the maximum conversion was reached earlier (after 120 min) and at a lower temperature than in the case of the Pd/NiFe_2_O_4_-N-BCNT system. With respect to the aniline yield, a similar trend was observed; the aniline yield reached the maximum value at a lower temperature (at 293 K and 303 K) after only 120 min in the case of the carbon-nanolayer-supported sample (Figure 9B,D). Despite the lower nickel ferrite and palladium content (Table 2), the carbon-nanolayer-supported catalysts showed higher activity in aniline synthesis than the Pd/NiFe_2_O_4_-N-BCNT sample. Moreover, the ratio of the pyridinic nitrogen formed in the CNL was greater than that found in the N-BCNT structure (Table 1). This led to the formation of more vacancies (at high energy adsorption centrums) in the graphitic structure of the carbon nanolayer, which improved the anchoring of the palladium nanoparticles. Another important difference can also be observed with respect to the types of incorporated nitrogen forms in the structure of the CNL and N-BCNT, as the former also contained pyrrolic-type nitrogen (Table 1). The presence of the pyrrolic nitrogen in the structure of the carbon nanolayer contributed to the increased catalytic activity of the catalyst, because this type of nitrogen can form complexes with the Pd(II)-ions, which results in strong interaction between the catalytically active metal and its support. This was confirmed by Ombaka et al. in their experiments, in which pyrrolic nitrogen containing N-BCNT was tested as a catalyst support for palladium in the hydrogenation of nitrobenzophenone [43]. The catalytic activity and selectivity of the Pd/N-BCNT catalyst depended on the amount of pyrrolic nitrogen and not on the total nitrogen content. The increased activity was explained by improved interaction between the N-BCNT and Pd. By increasing the pyrrolic nitrogen content, better dispersion and more stable palladium nanoparticles were provided. Accordingly, it was concluded that a strong interaction formed between the pyrrolic nitrogen and Pd particles (confirmed by XPS measurements) because Pd^2+^ ions made Pd–N coordination complexes, which stabilized the palladium nanoparticles. Another contribution to the increased activity of the Pd/NiFe_2_O_4_-CNL was the morphological feature of the carbon nanolayer. On the carbon foils, the highly dispersed palladium nanoparticles formed continuous coverage, which was easily accessible to the reactant molecules.

**Figure 10:**
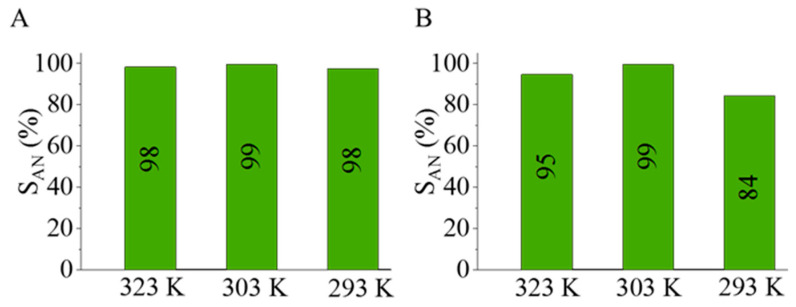
Selectivity of aniline after 240 min of hydrogenation using Pd/NiFe_2_O_4_-CNL (**A**) and Pd/NiFe_2_O_4_-N-BCNT (**B**).

The aniline selectivity of the Pd/NiFe_2_O_4_-CNL was higher than that the Pd/NiFe_2_O_4_-N-BCNT catalyst (Figure 10). In the case of the carbon-nanolayer-supported catalyst, the aniline selectivity was above 98 n/n% at each studied reaction temperature (at 293 K, 303 K, and 323 K), in contrast to the N-BCNT-supported sample, where the maximum selectivity was only 84 n/n% at 293 K. The synergistic effect of the pyrrolic-nitrogen-containing carbon nanolayer support also contributed to the increased aniline selectivity, which is similar to the findings of Ombaka et al. [43].

**Figure 11:**
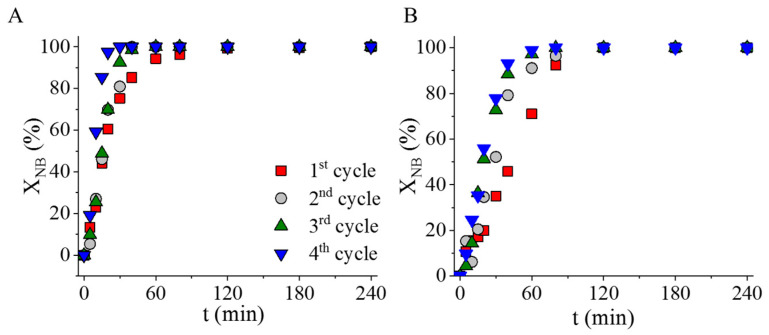
Reuse tests of the Pd/NiFe_2_O_4_-CNL (**A**) and Pd/NiFe_2_O_4_-N-BCNT (**B**) catalysts. Nitrobenzene conversion vs. time of hydrogenation.

The stability, namely, the strong interaction between the palladium and the carbon catalyst supports, was examined via reuse tests. The tests were carried out in four cycles of nitrobenzene hydrogenation at 323 K and 20 bar hydrogen pressure. The catalysts were not regenerated between cycles, but only rinsed with methanol. The two catalysts maintained their high catalytic activity over four cycles (Figure 11). In the case of the N-BCNT-supported catalyst, a non-significant decrease in the change in nitrobenzene conversion was observed in the fourth cycle (Figure 11B). For the sake of explanation, after the fourth cycle, the palladium content of the dried catalyst was measured using ICP-OES. In the case of the Pd/NiFe_2_O_4_-CNL catalyst, the initial palladium content was 3.98 wt%, which changed to 3.90 wt% after the reuse tests. In this sense, the catalyst was stable, because a strong interaction was formed between the catalytically active metal and its support. The situation was different in the case of the Pd/NiFe_2_O_4_-N-BCNT system because the initial palladium content of the fresh catalyst was 4.26 wt%, which decreased to 3.60 wt%, meaning that 0.66 wt% palladium was leached after four cycles. Based on this, it can be stated that the carbon nanolayer formed a stronger interaction with the palladium particles than the N-BCNT. This difference can be explained by the higher pyridinic nitrogen content, the presence of pyrrolic nitrogen, more negative electrokinetic potential, and the variety of oxygen-containing surface functional groups.

## 3. Materials and Methods

### 3.1. Materials

For the preparation of the carbon nanolayers (CNL), 4-nitroaniline (Alfa Aesar GmbH, Karlsruhe, Germany) and sulfuric acid (95 wt%, VWR Intern. S.A.S, Fontanay-sous-Bois, France) were applied. The activation of this sample was carried out by CO_2_ (Gourmet, Messer, Budapest, Hungary). The N-BCNTs were produced by the CCVD method from *n*-butylamine (Sigma Aldrich Ltd., 3050 Spruce Street, Saint Louis, MO, USA) as a carbon source. During CCVD synthesis of the carbon nanotubes, nickel(II) nitrate hexahydrate, Ni(NO_3_)_2_·6 H_2_O, MW: 290.79 g/mol (Thermo Fisher GmbH, Kandel, Germany) impregnated magnesium oxide (Merck KGaA, D-64271 Darmstadt, Germany) were employed as catalysts. For the preparation of the nickel–ferrite particles, iron(III) nitrate nonahydrate (Fe(NO_3_)_3_·9H_2_O, MW: 404.00 g/mol, VWR Int. LtD., Leuven, Belgium), ethylene glycol (HOCH_2_CH_2_OH, VWR Int. Ltd., Fontenay-sous-Bois, France), ethanolamine (NH_2_CH_2_OH, Merck KGaA, Darmstadt, Germany), and sodium acetate (CH_3_COONa, ThermoFisher GmbH, Kandel, Germany) were used. Palladium(II) nitrate dihydrate (Pd(NO_3_)_2_*2H_2_O, Alfa Aesar LtD., Ward Hill, MA, USA) was applied to deposit Pd onto the carbon supports. Nitrogen (purity 4.0, Messer) and hydrogen (purity 4.0, Messer) were used during the experiments. Nitrobenzene (Acros Organics, New Jersey, NJ, USA) was used as a reactant during the catalytic hydrogenation tests. The applied analytical standards (azobenzene, nitrosobenzene, *N*-methylaniline) were obtained from Sigma-Aldrich Co. (St. Louis, MO, USA).

### 3.2. Characterization Techniques

The morphology and structure of the carbon nanostructures were characterized using a high-resolution scanning electron microscope (SEM), applying a Helios G4 PFIB CXe (Thermo Scientific) instrument and using carbon tape for sample preparation. High-resolution transmission electron microscopy (HRTEM, Talos F200X G2 electron microscope, Thermo Fisher Scientific Inc., Budapest, Hungary) with field emission electron gun, X-FEG, accelerating voltage: 20–200 kV) was used for the characterization of particle size and morphology in the case of N-BCNT, carbon nanolayer, ferrite, and palladium nanoparticle samples. For the imaging and electron diffraction, a SmartCam digital search camera (Ceta 16 Mpixel, 4 k × 4 k CMOS camera) and a high-angle annular dark-field (HAADF) detector were used. Sample preparation was carried out from the aqueous dispersion of the nanoparticles by dropping them onto 300 mesh copper grids (Ted Pella Inc., Redding, CA, USA). For the qualitative and quantitative analysis of the samples, X-ray diffraction (XRD) was employed. The Bruker D8 diffractometer (Bruker, Karlsruhe, Germany) was equipped with a Cu-Kα source, a Göbel mirror in parallel beam geometry, and a Vantec detector. The average crystallite size was calculated via the mean column length calibrated method by using full width at half maximum (FWHM) and the width of the Lorentzian component of the fitted profiles. The zeta potential of the ground carbon nanostructures was measured in the aqueous phase by determining the electrophoretic mobility of the particles (laser Doppler electrophoresis) using a Malvern Zetasizer Nano ZS (Malvern, Worcestershire, United Kingdom). The palladium content of the catalysts was measured by a Varian 720 ES (Varian, Palo Alto, California, United States) inductively coupled optical emission spectrometer (ICP-OES). For the ICP-OES measurements, the samples were dissolved in hydrochloric and nitric acid. The specific surface area (SSA) analysis was carried out using the carbon-dioxide adsorption–desorption method at a temperature of 273 K. For the measurements, Micromeritics ASAP 2020 (Micromeritics, Norcross, GA, USA) equipment was used, and then the evaluation was carried out based on the Dubinin–Astakhov (in the case of Pd/NiFe_2_O_4_-N-BCNT) or the Dubinin–Radushkevich (Pd/NiFe_2_O_4_-CNL) method. The incorporated nitrogen and the oxygen-containing functional groups were identified using X-ray photoelectron spectroscopy (XPS). During the measurements, a Kratos XSAM-800 XPS (Kratos Analytical, Manchester, United Kingdoms) instrument was utilized with a MgKα X-ray source operated at 120 W (12 kV, 10 mA). Samples were carefully mounted on double-sided carbon tape, paying attention to the consistent coverage of the holder. Survey spectra were collected with a pass energy of 80 eV and a 1 eV step size. High resolution spectra (C 1s, N 1s) were collected with a pass energy of 40 eV and a 0.1 eV step size. Gas chromatography (GC) was used to measure the quantity of nitrobenzene, aniline, and the intermediate and side-products. For the measurements, an Agilent7890A (Agilent, Santa Clara, CA, USA) instrument equipped with an RTX 624 column (60 m × 0.25 m × 1.4 µm) was used, in addition to an Agilent 5975C mass spectroscope. For the samples, a 200:1 split was used and the column was heated to 200 °C.

### 3.3. Preparation of the Carbon Nanolayer (CNL) Sample

4-Nitroaniline (1.50 g) powder and 1 mL of concentrated (95 wt%) sulfuric acid were mixed in a ceramic crucible. The mixture was heated using a Bunsen burner to carbonize 4-nitroaniline. The formed carbon foams were washed in distilled water five times, and the purified sample was dried at 378 K overnight. The activation treatment was carried out in two steps: first, the sample was heated at 673 K for 30 min under nitrogen flow; then, it was heated at 1173 K for another 30 min in a carbon dioxide atmosphere.

### 3.4. Preparation of the N-BCNT

The 5 wt% nickel-containing magnesium oxide was placed into a quartz reactor in a tube furnace, which was anellated at a temperature of 1023 K. The carbon source (butylamine) was dosed via a syringe pump (6 mL h^−1^) into the reactor, where the source formed vapor and flowed together with the nitrogen (100 mL min^−1^) into the catalyst bed. The nickel-containing catalyst was removed from the nanotubes with hydrochloride acid. The purity of the N-BCNT sample was determined by thermogravimetric analysis.

### 3.5. Deposition of the Nickel–Ferrite Particles onto the Surfaces of the Carbon Supports

Amine-functionalized ferrite magnetic catalyst supports were synthesized by applying a modified coprecipitation method. In 400 mL ethylene glycol, 32.32 g iron(III) nitrate nonahydrate and 11.63 g nickel(II) nitrate hexahydrate were dissolved. In the other mixture, 49.22 g sodium acetate was solved in 2000 mL ethylene glycol, and 10 g carbon (N-BCNT or CNL) was dispersed by a Hielscher UIP 1000 hDT ultrahigh efficient homogenizer. The dispersion was heated to 373 K in a round-bottom flask under reflux with continuous stirring. The metal-ion-containing solution was added into the glycol-based carbon-containing dispersion, and 140 mL ethanolamine was also added to the mixture. After 12 h of continuous agitation and reflux, the cooled solution was separated by centrifugation (4200 rpm, 10 min). The solid phase was washed with distilled water several times, and the magnetic ferrite was easily separated by a magnet from the aqueous phase. Finally, the ferrite-decorated carbon samples were rinsed with absolute ethanol and dried at 353 K overnight. The produced ferrite-containing carbon samples were used as magnetic catalyst supports for the preparation of palladium-decorated spinel catalysts.

### 3.6. Deposition of the Palladium Nanoparticles onto the Surfaces of the Ferrite-Decorated Carbon Supports

The nickel–ferrite-decorated carbon support (2.00 g) was dispersed in 200 mL ethanol by ultrasonication using Hielscher UIP1000 hDT homogenizer. For the carbon dispersion, 50 mmol ethanolic solution of palladium(II) nitrate hydrate was added, then treated with ultrasonication for 10 min. The alcohol was evaporated by a rotary vacuum evaporator, and the catalysts were dried at 378 K overnight.

### 3.7. Catalytic Tests of the Prepared Palladium Catalysts

The hydrogenation of nitrobenzene in methanolic solution was carried out in order to study the catalytic activity of the magnetic, separable, carbon-supported palladium nanocomposites. The concentration of nitrobenzene was 0.25 mol·L^−1^ (150 mL), and 0.1 g catalyst was added to the system. The reaction was carried out in a Büchi Uster Picoclave reactor, which has a 200 mL stainless steel vessel with a heating jacket. The pressure of H_2_ was kept at 20 bar, and the reactants were thermostated at 293 K, 303 K, and 323 K. Sampling was carried out after 5, 10, 15, 20, 30, 40, 60, 80, 120, 180, and 240 min. The samples were measured by gas chromatography. The efficiency of the catalyst was characterized by calculating the conversion (*X*%) of nitrobenzene based on the following equation (Equation (1)):

(1) $$X\%=\frac{\;\;n_{consumed\;NB}}{n_{initial\;NB}}\;\cdot \;100\;$$

The aniline (*AN*) yield (*Y*%) was also calculated as follows (Equation (2)):

(2) $$Y\%=\frac{{n\;}_{formed\;AN\;}}{n_{\;theoritical\;AN\;}}\;\cdot \;100$$

Furthermore, *AN* selectivity (*S*%) was calculated according to the following equation (Equation (3)):

(3) $$S\%=\frac{Y}{X}\cdot \;100$$

## 4. Conclusions

Two nitrogen-doped nanostructured carbon materials were prepared and tested as catalyst supports. The CNL support was built up by a membrane-like foil structure stretched out between a carbon frame, which is an ideal, easily accessible surface for catalytically active metals. The N-BCNT support provided a mesoporous, bamboo-like structure with edges containing structural defects. These properties improved the catalytic activity of the catalyst due to the potential electron transfer from the metals to the electronegative atoms (e.g., C, N, O) of the support. Furthermore, nitrogen doping improved the materials’ physical properties (e.g., adsorption capacity or wettability) by creating surface defects in the structures. The developed nitrogen-doped, carbon-supported palladium catalysts were decorated by nickel–ferrite nanospheres to combine the aforementioned positive features with magnetic properties, and, thus, to create magnetizable catalysts which are easily separable by a magnetic field from the liquid phase reaction media without loss. Furthermore, the conventional, time-consuming separation steps (filtration, centrifugation) can also be avoided. The incorporation of pyridinic and pyrrolic nitrogen atoms into the system led to strong interaction between the palladium nanoparticles and the carbon supports; thus, these catalysts are reusable multiple times without regeneration and retain their exceptionally high catalytic activity (100% nitrobenzene conversion even after four test cycles lasting 120 min at 333 K), selectivity (98% for Pd/NiFe_2_O_4_-CNL and 95% for Pd/NiFe_2_O_4_-N-BCNT), and stability. Additionally, in the case of the Pd/NiFe_2_O_4_-CNL catalyst, no significant precious metal leaching was experienced compared to its Pd/NiFe_2_O_4_-N-BCNT counterpart (0.08 wt% vs. 0.66 wt%, respectively). In summary, both the Pd/NiFe_2_O_4_-CNL and Pd/NiFe_2_O_4_-N-BCNT catalysts are suitable to be employed in nitrobenzene hydrogenation, although the former is more preferable due to its higher stability.

## Figures and Tables

**Table 1:** Atomic percentages of the different incorporated nitrogen types.

Sample	Atomic Percentage of Nitrogen (%)			
	Pyridinic N	Graphitic N	Pyrrolic N	Oxidized N
CNL	43.6	15	32.2	9.2
N-BCNT	31.2	35.7	-	33.1

**Table 2:** Phase composition of the magnetic palladium catalysts.

wt%	NiFe_2_O_4_	Fe_3_O_4_	Ni	Pd
Pd/NiFe_2_O_4_-CNL	13.9	0.3	0.3	3.98
Pd/NiFe_2_O_4_-N-BCNT	19.6	5.3	1.0	4.26

## Data Availability

Data are available upon request from the corresponding authors.

## Supplementary Materials

The following supporting information can be downloaded at: https://www.mdpi.com/article/10.3390/ijms242417547/s1.
