# Supplementary material for: Development of Magnetizable, Nickel–Ferrite-Decorated Carbon Nanocomposites as Hydrogenation Catalyst for Aniline Synthesis
Source: Int J Mol Sci. 2023 Dec 16;24(24):17547. doi: 10.3390/ijms242417547 (PMC10743656; doi:10.3390/ijms242417547)
Supplement: Supplementary file 1 [file ijms-24-17547-s001.zip › ijms-2751898-supplementary.pdf]

Development of Magnetizable, Nickel–Ferrite-Decorated Carbon Nanocomposites as Hydrogenation Catalyst for Aniline Synthesis

Ádám Prekob [1], Máté Péter Szegedi [1], Gábor Muránszky [1], Ferenc Kristály [2], Miklós Nagy [1], Gyula Halasi [3,4], Ákos Szamosvölgyi [3], Béla Fiser [5,6,7], Béla Viskolcz [1,5] and László Vanyorek [1,*]

[1] Institute of Chemistry, University of Miskolc, Miskolc-Egyetemváros, 3515 Miskolc, Hungary; adam.prekob@uni-miskolc.hu (Á.P.); mate.peter.szegedi@uni-miskolc.hu (M.P.S.); gabor.muranszky@uni-miskolc.hu (G.M.); miklos.nagy@uni-miskolc.hu (M.N.); bela.viskolcz@uni-miskolc.hu (B.V.)
[2] Institute of Mineralogy and Geology, University of Miskolc, Miskolc-Egyetemváros, 3515 Miskolc, Hungary; askkf@uni-miskolc.hu
[3] Department of Applied and Environmental Chemistry, University of Szeged, Rerrich Béla Square 1., 6720 Szeged, Hungary; halasigy@chem.u-szeged.hu (G.H.); szamosvolgyi@chem.u-szeged.hu (Á.S.)
[4] ELI-ALPS, ELI-HU Non-Profit Ltd., Wolfgang Sandner Utca 3., H-6728 Szeged, Hungary
[5] Higher Education and Industrial Cooperation Centre, University of Miskolc, Miskolc-Egyetemváros, 3515 Miskolc, Hungary; bela.fiser@uni-miskolc.hu
[6] Ferenc Rakoczi II Transcarpathian Hungarian College of Higher Education, 90200 Beregszász, Ukraine
[7] Department of Physical Chemistry, Faculty of Chemistry, University of Lodz, 90-236 Lodz, Poland
* Correspondence: laszlo.vanyorek@uni-miskolc.hu

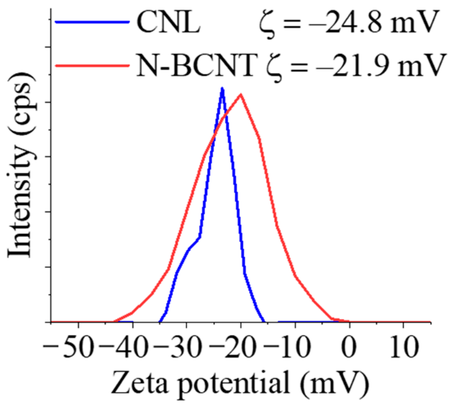

Figure S1: Zeta potential distribution of the CSA and N-BCNT catalyst supports

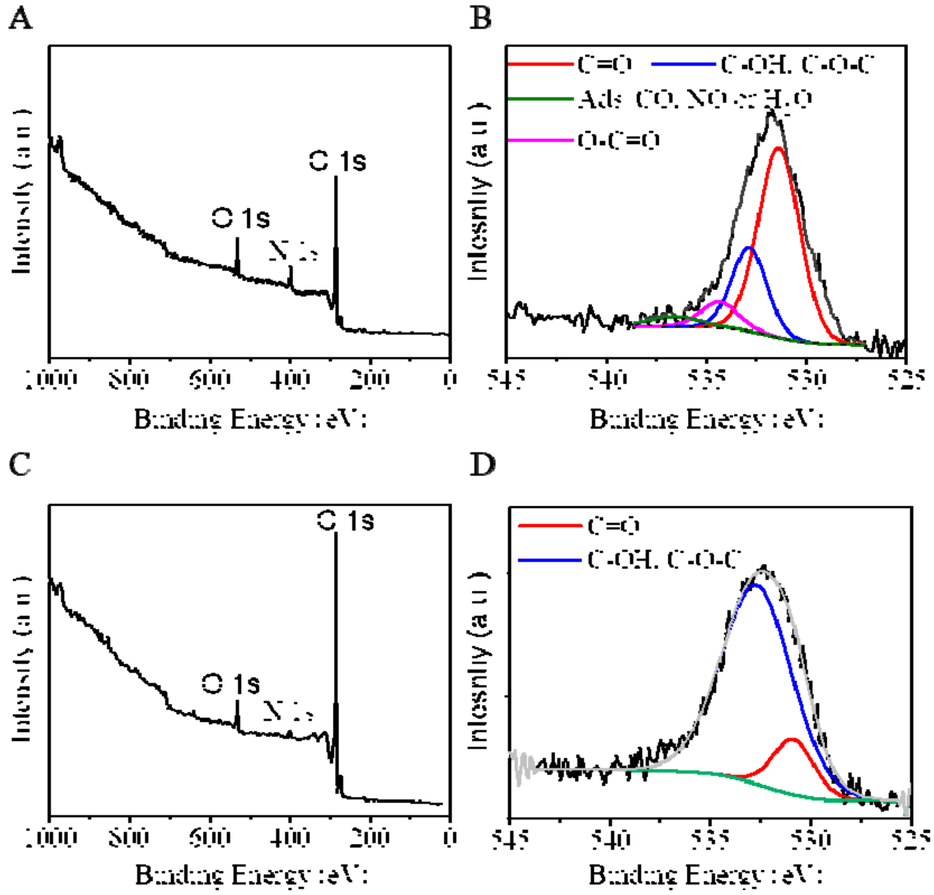

Figure S2: The Survey and O1s spectra of CNL (A, B) and N-BCNT (C, D)

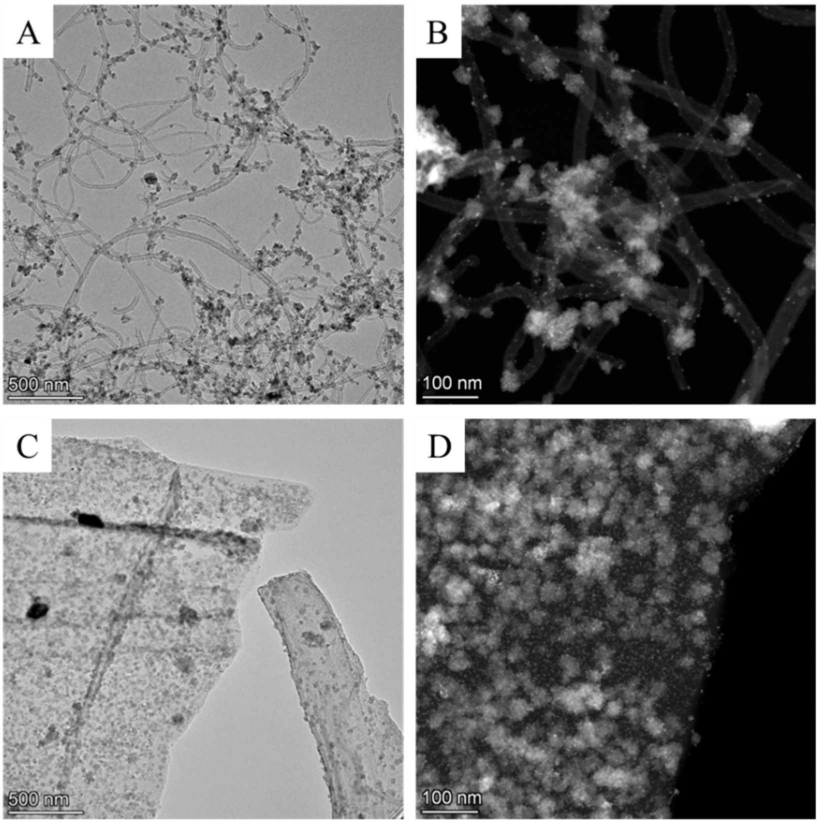

Figure S3: HRTEM and HRTEM-HAADF images of the Pd/$NiFe_2O_4$-N-BCNT (A, B) and Pd/$NiFe_2O_4$-CNL (C, D) systems

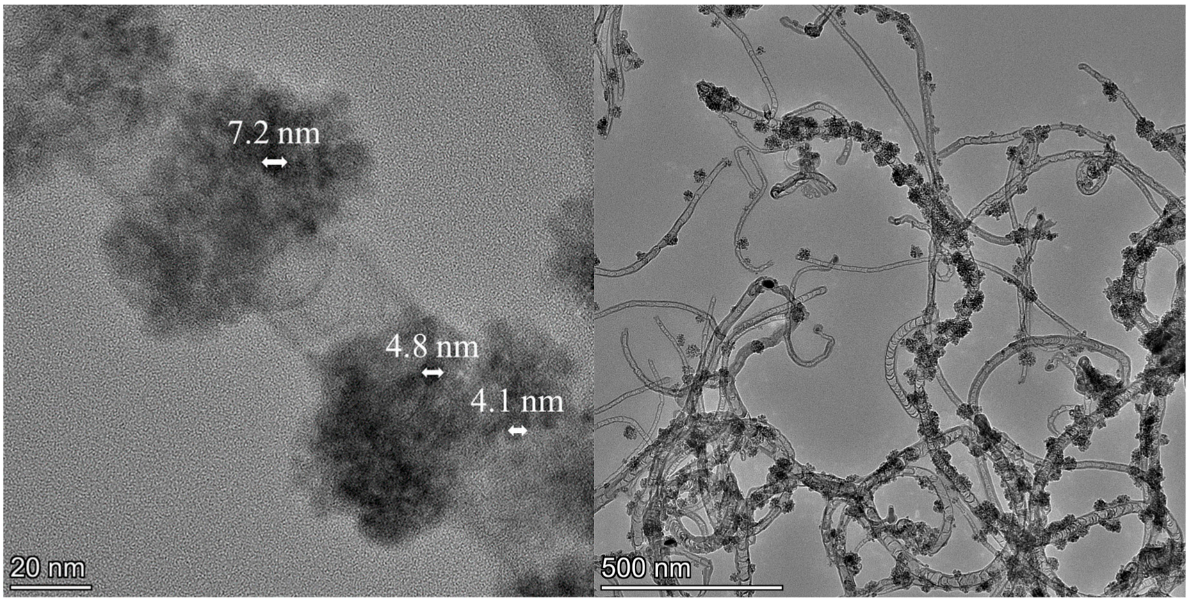

Figure S4: HRTEM image of $NiFe_2O_4$ nanoparticles on the surface of N-BCNT support

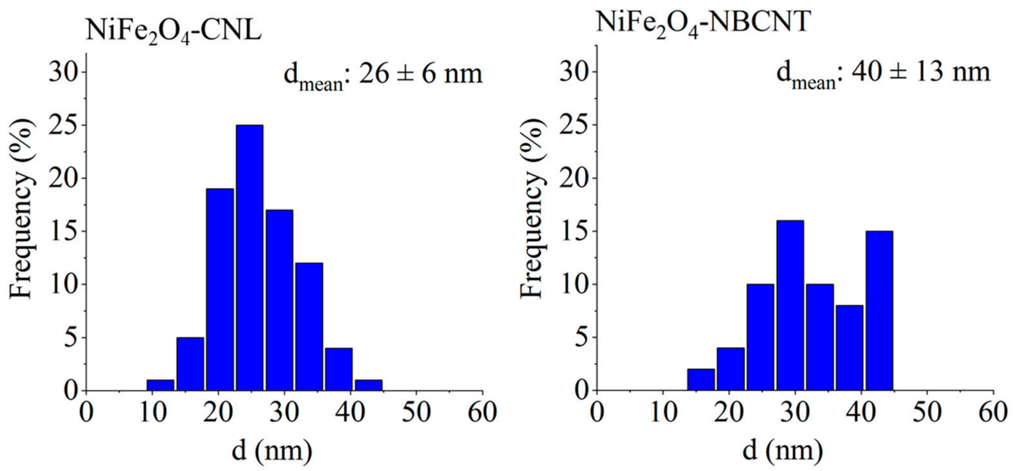

Figure S5: Particle size distribution of the $NiFe_2O_4$ nanospheres on the CNL and N-BCNT supports

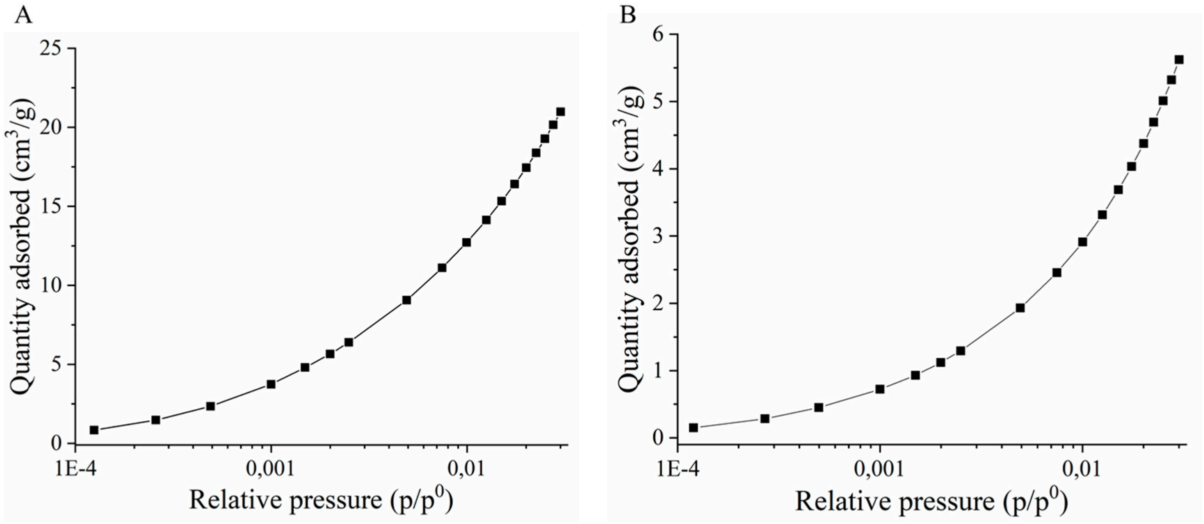

Figure S6: Sorption isotherms of Pd/$NiFe_2O_4$-CNL (A) and Pd/$NiFe_2O_4$-N-BCNT (B) samples
